# Evidence for excessive osteoclast activation in SIRT6 null mice

**DOI:** 10.1038/s41598-018-28716-z

**Published:** 2018-07-20

**Authors:** Demao Zhang, Junjun Jing, Feng Lou, Ruimin Li, Yilin Ping, Fanyuan Yu, Fanzi Wu, Xiao Yang, Ruoshi Xu, Feifei Li, Ke Wang, Mingru Bai, Caixia Pi, Jing Xie, Liwei Zheng, Ling Ye, Xuedong Zhou

**Affiliations:** 1 State Key Laboratory of Oral Diseases, National Clinical Research Center for Oral Diseases, West China Hospital of Stomatology, Sichuan University, Chengdu, China; 2 Department of Endodontics, Stomatology Hospital, General Hospital of NingXia Medical University, Yinchuan, China; 3 Department of Stomatology, Tongde Hospital of Zhejiang Province, Zhejiang, China; 4 Department of Biomedical Sciences, Texas A&M College of Dentistry, Dallas, TX USA

## Abstract

SIRT6 is a NAD-dependent histone 3 deacetylase. SIRT6 null mice have been reported suffering osteopenia. However, the role of SIRT6 in bone resorption is still not well understood. In this study, we focused on the role of SIRT6 in osteoclast. We performed histological analysis on the femur, spine, alveolar bone and even tail of mutant mice, and found the bone mass is sharply decreased while the osteoclast activity is significantly increased. These phenotypes were further demonstrated by the osteoclast differentiation in cell-cultures with TRAP staining and Pit Resorption Assay. We next found the proliferation activity of mutant osteoclast precursors was increased, which might account for the enhanced osteoclast formation. The concentration of tartrate-resistant acid phosphatase 5b, a marker of osteoclast differentiation, was significantly higher in the mutant mice than control. Besides, the osteoclastogenic and NF-κB signaling related genes were significantly up-regulated. Moreover, osteoblast/osteoclast co-culture demonstrated that SIRT6 regulated osteoclast mainly through osteoblast paracrine manner, rather than osteoclast-autonomous behavior. Together, the enhanced osteoclast activation in SIRT6 null mice might be regulated by the hyperactive NF-κB signaling and the enhanced proliferation activity of osteoclast precursors through osteoblast paracrine manner at the cellular level.

## Introduction

Osteoporosis, featured as dramatic bone loss, is a bone disease that mostly happens in elderly people due to unbalance of bone homeostasis^1,2^. Bone mass is maintained through the coordinated processes of different bone cells. Osteoblasts are the cells responsible for bone formation while osteoclasts are the cells involved in bone resorption. These cells produce factors that stimulate intercellular signaling, and strictly regulate bone formation and resorption to achieve bone homeostasis^3,4^. Osteoporosis could be developed by either insufficient bone formation or excessive bone resorption, which corresponds to retarded osteoblast or hyperactive osteoclast, respectively^1,2,5^. Thus, the hyperactive osteoclast activation is critical for the development of osteopenia^3,6,7^.

SIRT6 is one of seven mammalian Sirtuin family members, designated as SIRT1–SIRT7. And SIRT6 is a NAD^+^-dependent histone 3 deacetylase and classified into the class III histone deacetylases (HDACs) family^8^. SIRT6 is involved in various nuclear actions, including telomeric chromatin maintenance, genome stabilization, DNA repair and gene expression programs^9^. Recent studies have revealed that SIRT6 has multiple functions in the regulation of inflammation and metabolism by suppressing nuclear factor kappa B (NF-κB) target molecules via interaction with the RelA subunit of NF-κB^10,11^. Gain and loss function of SIRT6 has revealed SIRT6 could regulate bone formation via impacting osteoblast differentiation^12^. SIRT6 knockout mice suffered a progeroid degenerative syndrome including osteopenia, which showed 30% bone loss compared with the littermates of wild type^9,13^. Apparently, bone loss was the overall effects of abnormal bone formation and/or resorption caused by osteoblast and/or osteoclast defects^13^.

In this study, we focused on the role of SIRT6 in regulation of osteoclast. It was reported that overexpression of SIRT6 could suppress inflammatory responses and protect bone destruction in mice via reducing osteoclast formation. Bone marrow-derived monocyte/macrophage precursors cells (BMMs) with SIRT6 overexpression was confirmed to show less osteoclast formation^10,11^. While SIRT6 deficiency led to more osteoclast differentiation^14^. In contrast, it was also discrepantly reported that Sirt6 deficiency led to decreased osteoclast differentiation^13,15^. Therefore, it remains unclear how SIRT6 regulates osteoclast differentiation and bone resorption. In this study, we analyzed the femur, spine, alveolar bone and tail of SIRT6 null mice, and found that bone mass was sharply decreased while osteoclast activation was significantly increased, which were further demonstrated by osteoclast cell-cultures of differentiation and function with TRAP staining (Tartrate-resistant acid phosphatase) and Pit Resorption Assay, respectively. Additionally, we found that SIRT6 deficiency promoted the proliferation of osteoclast precursors at the early stage of cell-culture *in vitro*, which might partially lead to the enhanced osteoclast formation. We also detected the concentration of osteoclast differentiation in mouse serum, and found the concentration of tartrate-resistant acid phosphatase 5b(ACP5) was significantly higher in the mutant mice, compared with the littermates of wild type. We further found that the osteoclastogenic genes and NF-κB signaling related genes were upregulated, which were consistent with the previous related works^10,12,16,17^. Moreover, in order to better understand the cellular behaver of SIRT6 null osteoclast *in vivo*, we performed osteoblast/osteoclast differentiation co-culture, and demonstrated that the way SIRT6 regulated osteoclast was mainly through osteoblast paracrine manner, rather than osteoclast-autonomous behavior. Our work provided a new insight into the pathogenesis of osteoporosis due to SIRT6 mutation, and might be helpful to find the potential strategy of therapy.

## Results

### SIRT6 deficiency led to skeletal defects and bone loss

**Figure 1:**
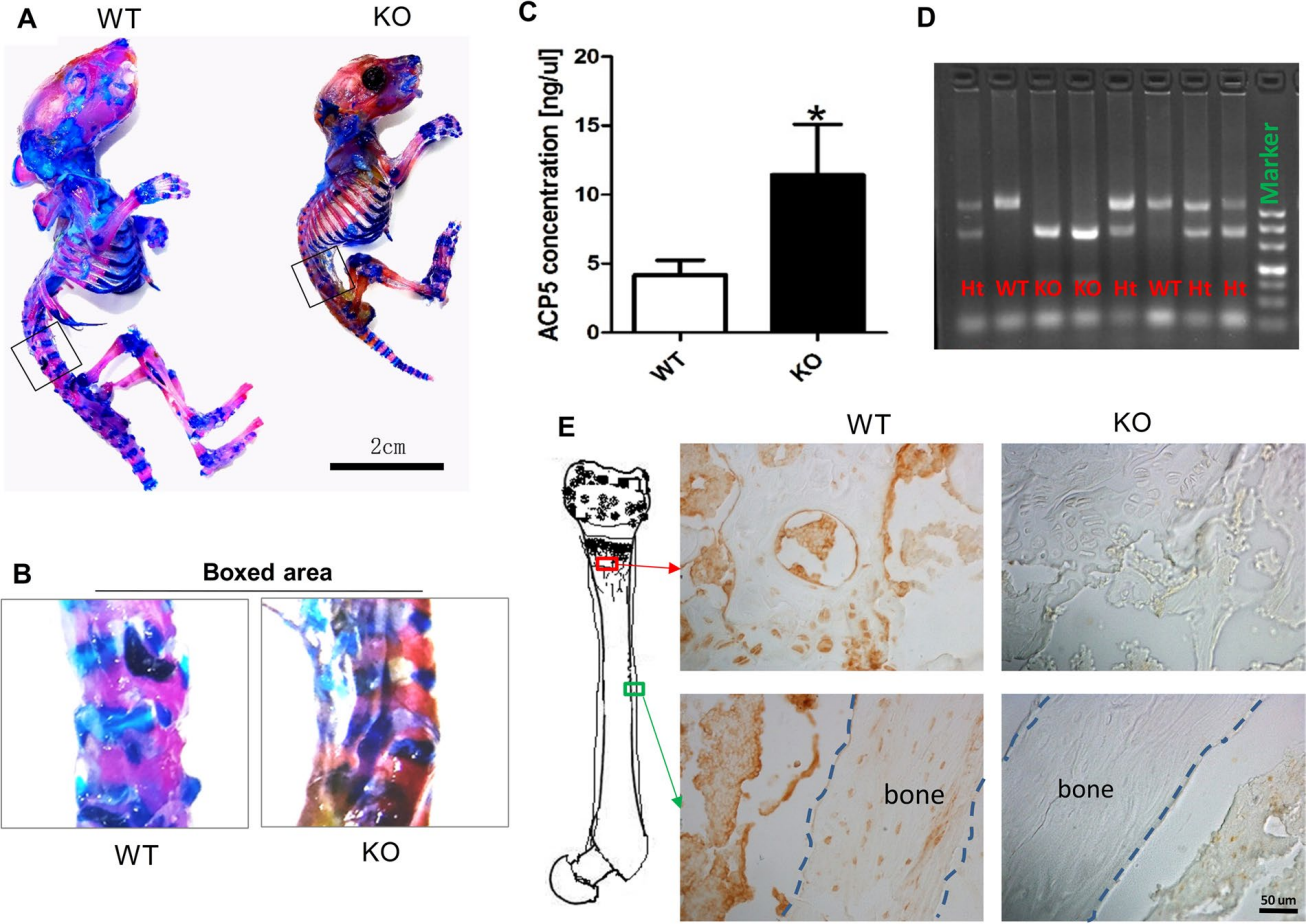
SIRT6 deficiency resulted in skeletal defects along with higher concentration of osteoclast differentiation marker in SIRT6 null mice. (**A**) Alcian Blue-Alizarin Red Skeletal Staining showed dwarf and skeletal defects of SIRT6 null mice compared with its wild type littermates, at 3-week old. (**B**) The boxed area showed the size change of lumbar vertebra between WT and SIRT6 KO mice. (**C**) ELISA showed the concentration change of ACP5 in the mouse serum, a marker of osteoclast differentiation. The number of mice in each group was about 9~11. *P < 0.05. (**D**) Genotyping of SIRT6 for the littermates of mice. Ht = Heterozygote, WT = Wild Type, KO = Knockout. (**E**) Immunohistochemistry confirmed SIRT6 was completely knocked out in the mutant mice.

To analyze the condition of bone homeostasis in SIRT6 null mice, conventional homozygous SIRT6 knockout mice (KO) were generated by crossing heterozygous mouse pairs. Alcian Blue-Alizarin Red Skeletal Staining showed the dwarf body size and skeletal defects of knockout mice compared with the wild type littermates (Fig. 1A). Both of the calcified bone (red) and the cartilage (blue) were shorter and shown to be abnormal morphology. We also detected the shorter vertebral body (VB) in lumbar vertebra and smaller cartilage layer around intervertebral disk (Fig. 1B). Meanwhile, we examined the concentration level of ACP5 in mouse serum and found it was significantly increased (up to about 280%) (Fig. 1C), which suggested the osteoclast differentiation could be overactive in SIRT6 KO mice. We divided the littermates into the WT and KO groups based on the genotypes (Fig. 1D). And we confirmed the SIRT6 expression in the KO group was really diminished both in trabecular bone and cortical bone of femur by immunostaining (Fig. 1E).

**Figure 2:**
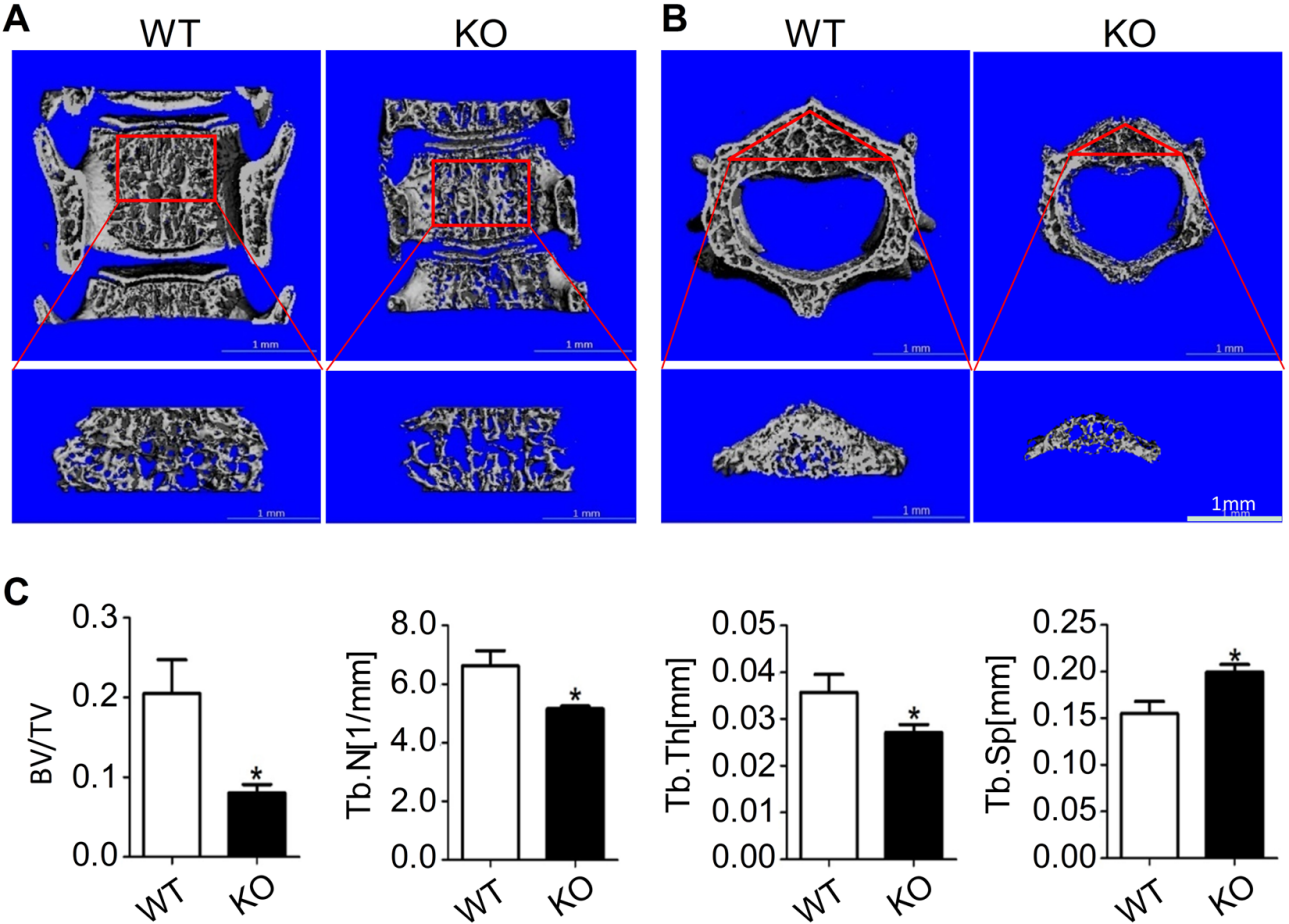
SIRT6 deficiency resulted in bone loss. (**A**) μ-CT scan showed the bone mass change of vertebral body from longitudinal view. (**B**) μ-CT scan showed the bone mass change of vertebral body from transversal view. (**C**) The main parameter of vertebral body changed. The number of mice in each group was about 5~7. BV/TV = Bone Volume/Total Volume; Tb. N = Trabecular Number; Tb. Th = Trabecular Thickness; Tb. Sp = Trabecular Separation/Spacing. *p < 0.05.

μ-CT scanning was performed for the bone histomorphometric analysis. It showed that SIRT6 knockout mice had a smaller vertebral body and less bone mass than control littermates. The results from longitudinal scan (Fig. 2A) and transverse scan (Fig. 2B) confirmed the bone loss in the SIRT6 null mice. And the alveolar bone of SIRT6 KO mice was significantly reduced as well (Fig. S2). From the analysis of μ-CT, we found that BV/TV, trabecular number and trabecular thickness were all significantly reduced (about 39%, 78% and 77%, respectively). The ratio of Trabecular Separation/Spacing (Tb.Sp) was resultantly increased up to about 129% (Fig. 2C). Together, SIRT6 deficiency resulted in obvious bone loss along with higher osteoclast differentiation activity in mice.

### SIRT6 deficiency increased osteoclast and decreased bone mass

**Figure 3:**
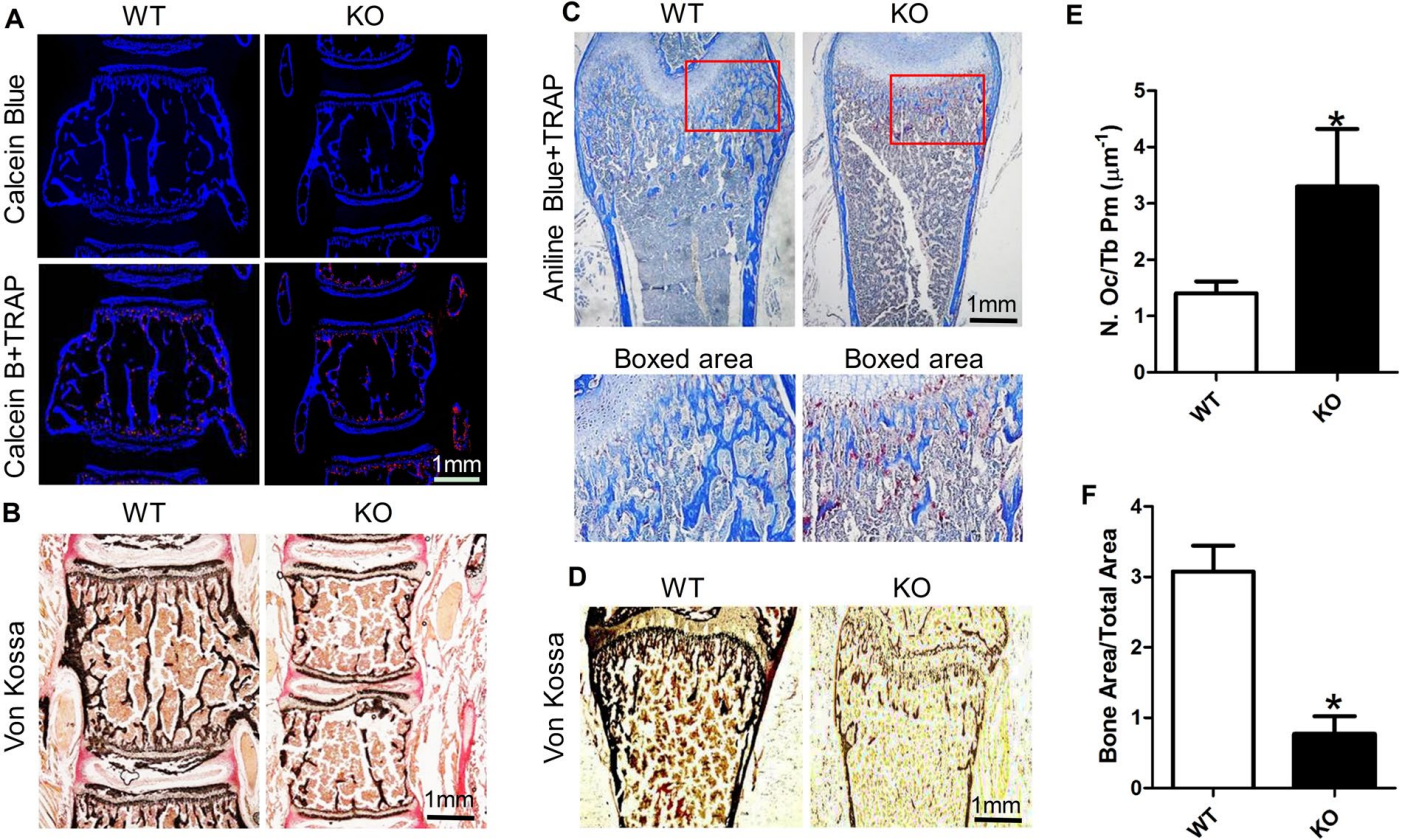
SIRT6 deficiency resulted in more osteoclasts and less bone mass in both spine and femur. (**A**) TRAP staining showed the osteoclast(red) increased in the vertebral body of KO group. Calcein Blue, the background staining for bone. (**B**) Von kossa staining further showed the decreased bone mass (black) in the vertebral body of the KO group. Van Gieson staining (red), the background staining. (**C**) TRAP staining showed the osteoclast (red) increased in the femur cancellous bone of KO group. Aniline Blue, the background staining (blue). (**D**) Von kossa staining showed the bone mass (black) decreased in the femur cancellous bone of KO group. (**E**) Statistical analysis showed the osteoclast changed by characterizing the ratio of the osteoclast number *vs*. the trabecular perimeter (N. Oc/Tb Pm). (**F**) Statistical analysis showed the bone mass changed by characterizing the ratio of bone area *vs*. total area, based on the Von Kossa staining. The number of mice in each group was about 3~5. *P < 0.05.

To further explore the mechanism of bone loss, we aimed to elucidate the histological changes in both lumbar vertebra and femur. By TRAP staining, we found the osteoclast number in lumbar vertebra of SIRT6 knockout mice was increased (Fig. 3A, red). Similarly, the osteoclast number in cancellous bone of femur was also increased and the cancellous bone mass was sharply reduced in the KO group (Fig. 3C, red). By Von Kossa staining, we further analyzed the bone mass in lumbar vertebra of SIRT6 knockout mice and found it was dramatically reduced (Fig. 3B). In femur, we also got the similar result to that in lumbar vertebra (Fig. 3D). Additionally, we found the bone mass in the tail of SIRT6 KO mice was sharply reduced along with increased osteoclasts (Fig. S1). By analyzing the histological data, we found the ratio of osteoclast number to trabecular perimeter (N. Oc/Tb Pm) was increased up to 235% (Fig. 3E), which verified the enhanced osteoclast in SIRT6 knockout mice. Meanwhile, statistical analysis showed the bone mass was significantly reduced by characterizing the ratio of the bone area *vs*. the total area based on the Von Kossa staining (Fig. 3F). Together, SIRT6 deficiency resulted in more osteoclasts with less bone mass in mice.

### The osteoclastogenic and NF-κB signaling genes were all increased in the SIRT6 null mice

**Figure 4:**
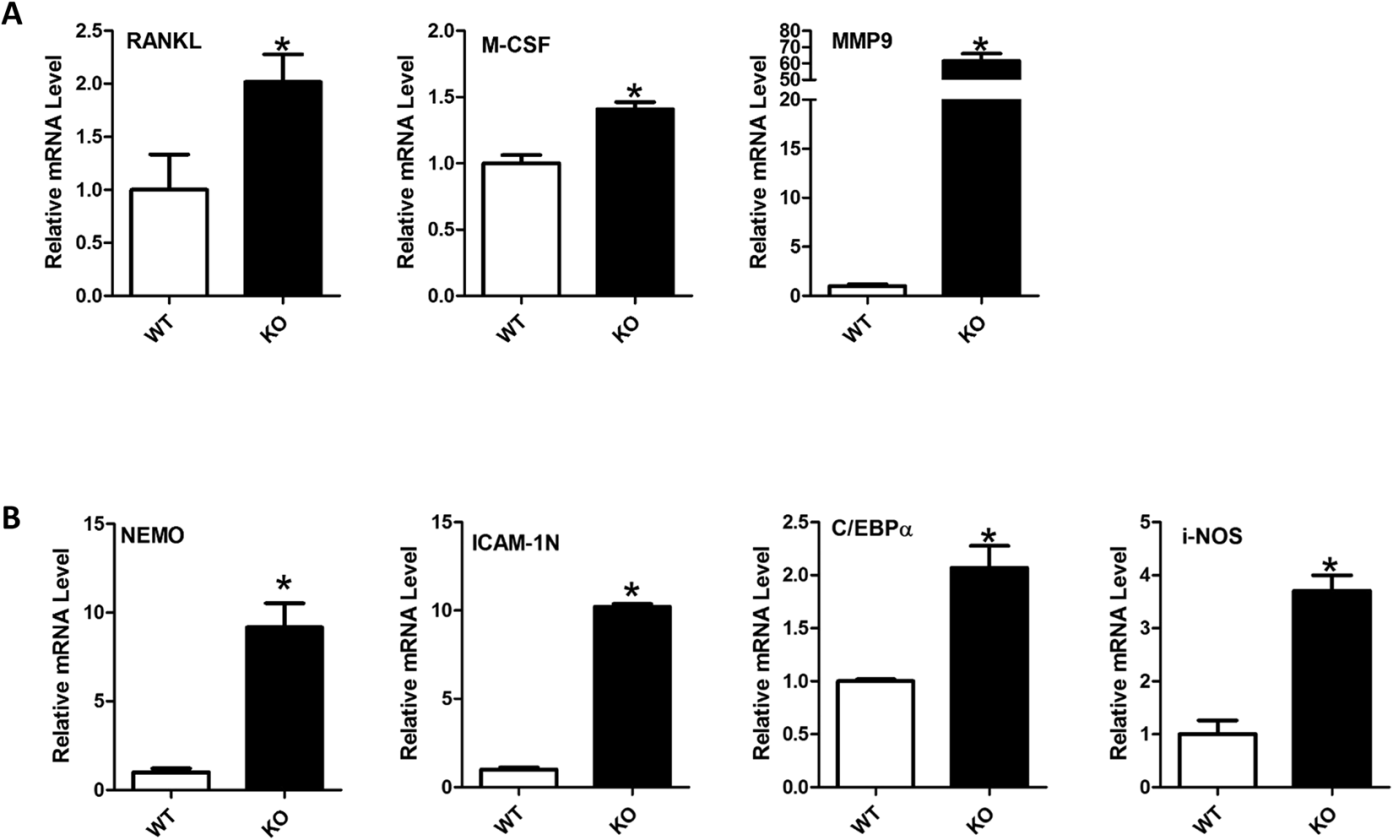
SIRT6 deficiency enhanced the expression of osteoclastogenic genes, as well as the NF-κB related genes in bone. (**A**) qPCR showed the mRNAs of osteoclastogenesis increased in the mutant group compared with the WT group (**B**) qPCR showed the mRNAs of NF-κB signaling increased in the mutant group compared with the WT group. The qPCR experiment data (**A**,**B**) were based on the samples of femurs. The data were the mean of three different experiments (n = 3). *p < 0.05.

To further identify the molecular mechanism of osteoclast change, we collected mRNA samples from long bone. By qPCR, we examined the mRNA changes of osteoclastogenesis and NF-κB signaling related genes, and the genes with significant different changes were showed in Fig. 4. For osteoclastogenic genes, we found the RANKL, MCSF and MMP-9 were significantly increased (Fig. 4A), which further confirmed the excessive activation of osteoclast in SIRT6 null mice at the molecular level. For NF-κB signaling related genes, we found the expression of NEMO, ICAM-1N, C/EBPα and i-NOS were elevated (Fig. 4B). Together, our data suggested SIRT6 deficiency promoted osteoclastogenesis likely through upregulating NF-κB signaling.

### SIRT6 deficiency promoted the formation of TRAP^+^ osteoclast, as well as the proliferation of osteoclast precursors

**Figure 5:**
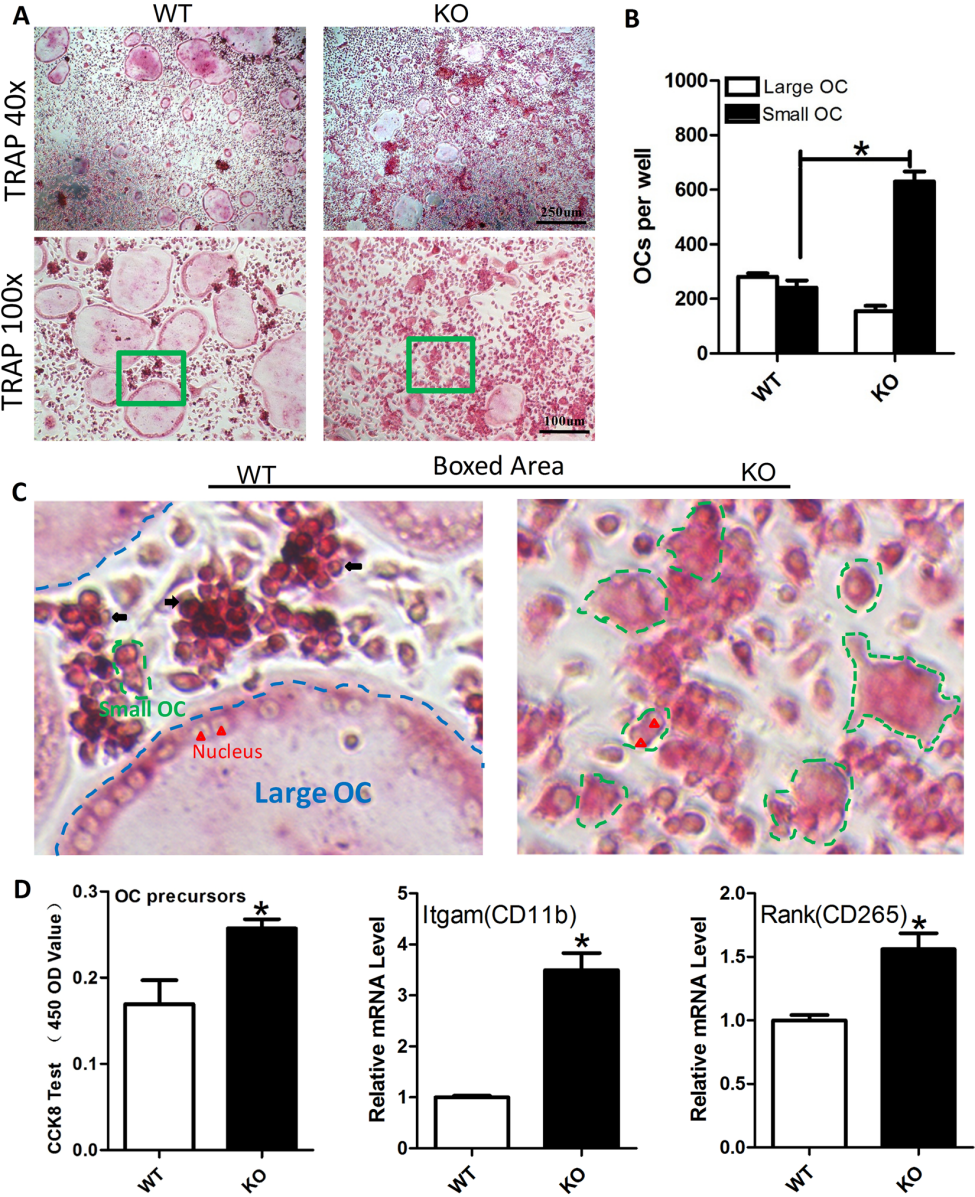
SIRT6 deficiency promoted the proliferation of osteoclast precursors, and increased the osteoclast formation. (**A**) TRAP staining showed the TRAP^+^ osteoclast increased in the KO group. (**B**) Statistical analysis showed the small osteoclast in the KO group significantly increased. (**C**) The small osteoclasts were increased in the KO group. In contrast, many of the small cells in the WT group were in the status of mononuclear cells without fusion (black arrow). Large OC (blue dotted line), osteoclast with many nuclei (red triangle) and a giant vacuole-like morphology. Small OC (green dotted line), TRAP^+^ osteoclast with multi-nucleus (cells had fused) but without vacuole-like morphology. (**D**) The proliferation of osteoclast precursors increased in the KO group, tested by CCK-8. And qPCR showed the expression of markers of osteoclast precursors, Itgam and Rank, increased in the bone marrow of KO mice *in vivo* (n = 3). *P < 0.05.

To further investigate the role of SIRT6 in osteoclast proliferation and differentiation, we cultured BMMs from bone marrow cavity of femur, and induced BMMs into osteoclast. After TRAP staining, we found that the large osteoclasts (the TRAP^+^ multinuclear-fused giant cells with vacuole-like morphology) were fewer in mutant mice compared with wildtype. However, the small osteoclasts (TRAP^+^ multinuclear-fused cells but without vacuole-like morphology) were significantly increased in the KO group (Fig. 5A). Indeed, the number of small osteoclasts in the KO group were significantly higher than that in the control group (Fig. 5B). In contrast, many small cells in the WT group were in the status of mononuclear cells without fusion (black arrow in Fig. 5C). Moreover, we analyzed the proliferation of osteoclast precursors at 24 h in the presence of MCSF by CCK-8 method, and found the proliferation was increased in the SIRT6 KO group (Fig. 5D). In addition, the expression of osteoclast precursors related markers, such as integrin alpha M (Itgam) and receptor activator of nuclear factor κB (Rank), were increased in the bone marrow of KO mice. Together, SIRT6 deficiency increased the formation of TRAP^+^ osteoclast, and it might be due to the enhanced proliferation activity of osteoclast precursors.

### SIRT6 deficiency functionally increased osteoclast activation

**Figure 6:**
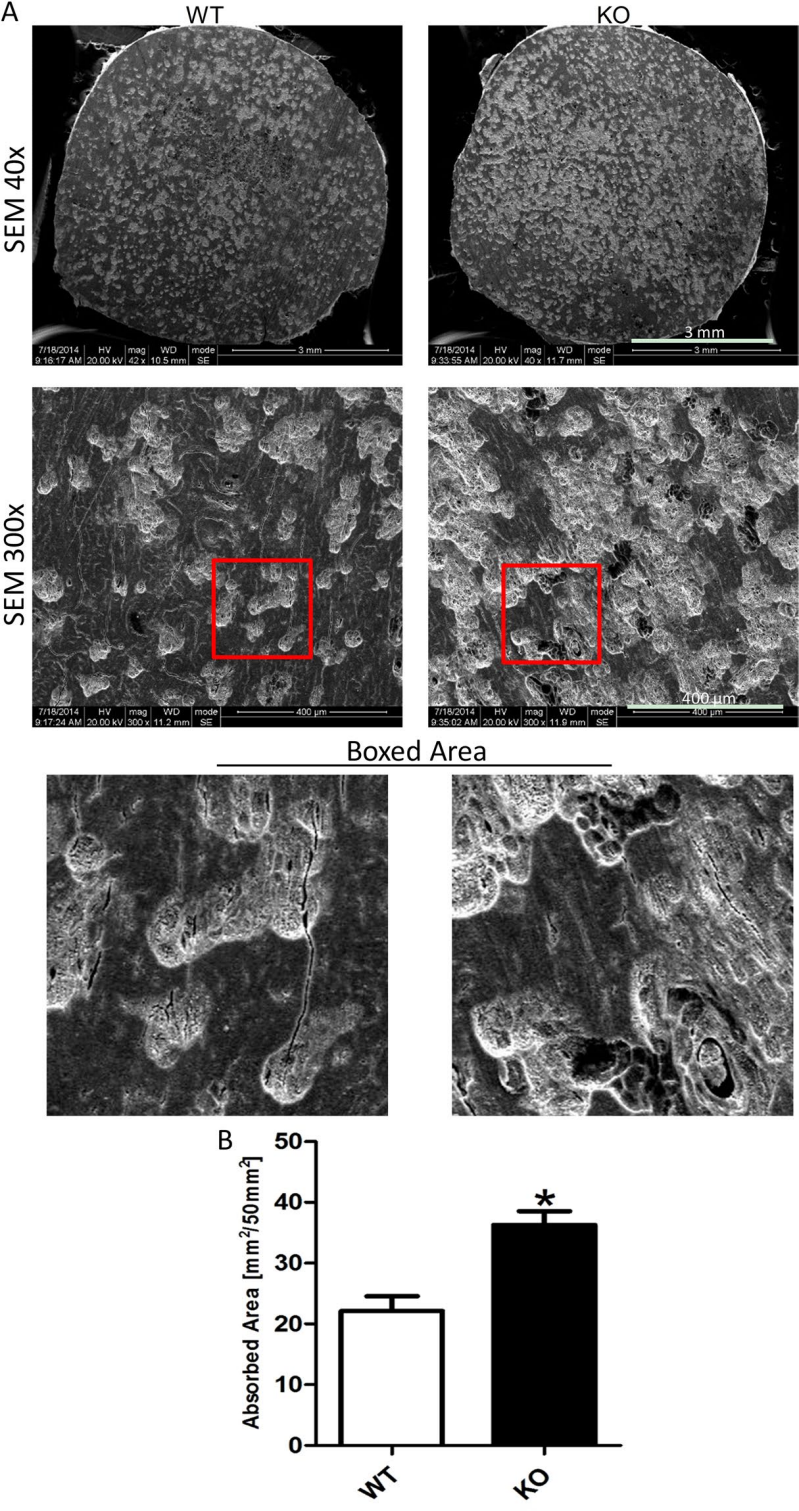
SIRT6 deficiency enhanced the osteoclast activation for bone resorption. (**A**) The bone resorption ability of mutant osteoclast increased, according to the Pit Resorption Assay via culturing the osteoclast on the slice of bovine cortical bone and tested by SEM (white area). (**B**) Statistical analysis showed the change of resorption area between the two groups. *p < 0.05.

To functionally examine the bone resorption ability of osteoclast, Pit Resorption Assay for osteoclast was performed. We found the osteoclast of SIRT6 knockout mice had the better bone resorption ability compared with the wild type mice, examined by scanning electron microscope (Fig. 6A), and the bone resorption area mostly doubled in the SIRT6 knockout mice compare with that in the wild type mice (Fig. 6B). Thus, this data confirmed the excessive activation of SIRT6 null osteoclasts, and consistent with the results above both *in vivo* and *in vitro*.

### The SIRT6 null osteoblast had a dominant role in promoting osteoclastogenesis

**Figure 7:**
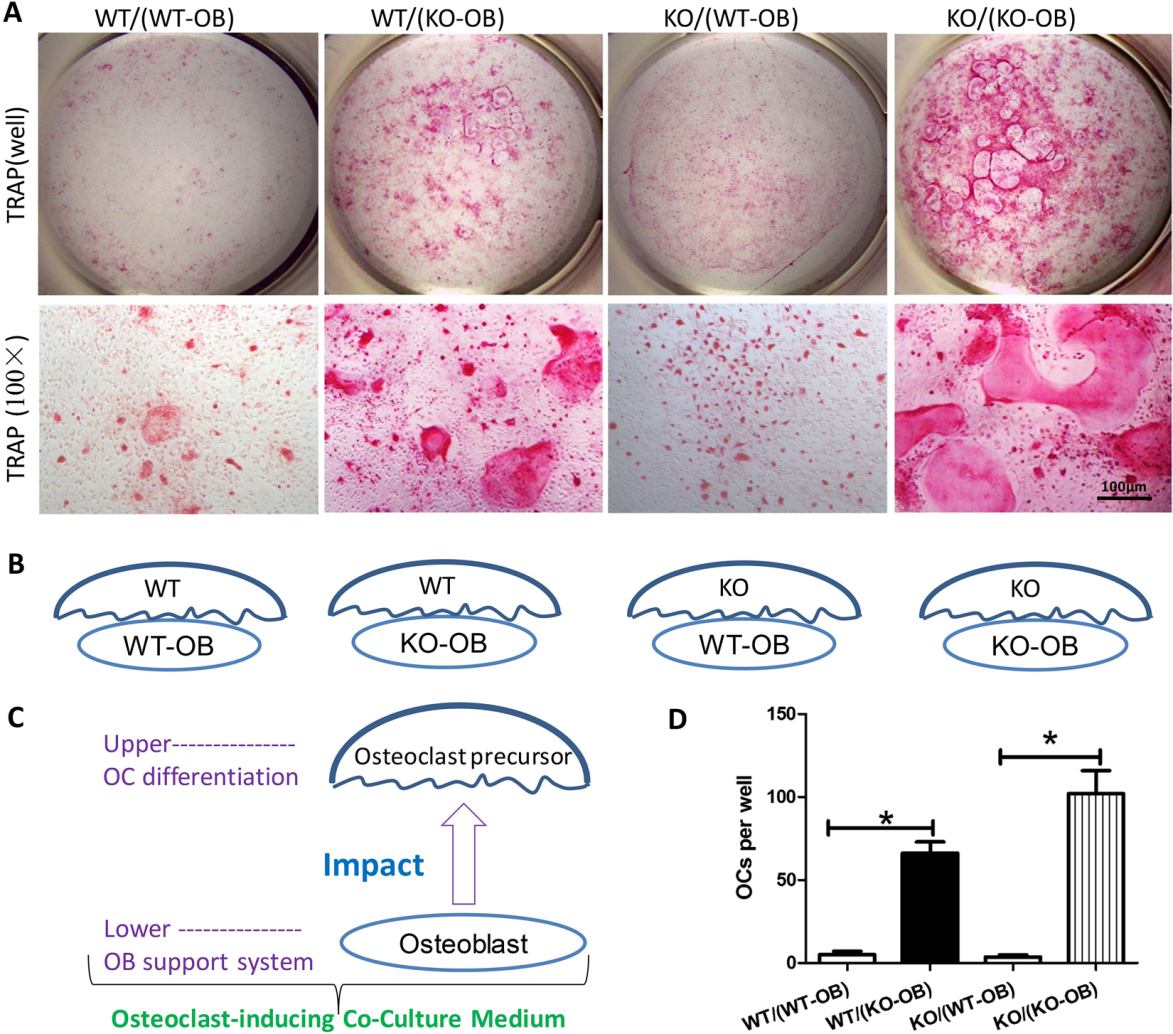
SIRT6 deficiency provoked osteoclast formation mainly through osteoblast paracrine manner. (**A**) TRAP staining for osteoblast/osteoclast co-culture showed more osteoclast formation when SIRT6 was deficient in osteoblast (WT/KO-OB or KO/KO-OB), rather than SIRT6 was deficient in osteoclast/osteoclast precursors. OC = osteoclast; OB = osteoblast. (**B**) and (**C**) The co-culture model indicated the group-dividing corresponding to the TRAP staining (**A**). The upper-layer cells were osteoclast precursors from BMMs for osteoclast differentiation, and the lower-layer cells, as osteoclast-inducing support system, were osteoblast/pre-osteoblast from neonatal calvaria. (**D**) Statistical analysis further showed the SIRT6 deficiency in osteoblast, not in osteoclast, made a dominant contribution to increasing osteoclast formation, by comparing WT/WT-OB to WT/KO-OB, as well as KO/WT-OB to KO/KO-OB (n = 3). *P < 0.05.

To better understand the cellular regulation mechanism of osteoclast by SIRT6, we performed osteoblast/osteoclast co-culture with primary cells of SIRT6 deficiency mice. TRAP staining revealed that the number of osteoclasts in the WT/KO-OB group was significantly higher compared with the WT/WT-OB group. Similarly, the number of osteoclasts in the KO/KO-OB group were also increased compared with the KO/WT-OB group (Fig. 7), suggesting the SIRT6 null osteoblast enhanced osteoclast differentiation. Statistical analysis (Fig. 7D) further confirmed that SIRT6 deficiency in osteoblast, not in osteoclast, made a dominant contribution to osteoclast formation, by comparing WT/WT-OB with WT/KO-OB, as well as KO/WT-OB with KO/KO-OB. Together, SIRT6 deficiency promoted osteoclast formation mainly through SIRT6 null osteoblast, rather than SIRT6 null osteoclast/osteoclast precursors.

## Discussion

An acute multiorgan degenerative syndrome occurred in SIRT6 null mice at 2 weeks after birth. The phenotypes included severe metabolic defects, spinal curvature abnormalities, lymphocyte attrition, loss of subcutaneous fat and low bone mineral density^9,16^. In this work, we further uncovered the skeletal defects of the SIRT6-null mice. The bone mass of femur, lumbar vertebra, alveolar bone and tail were significantly decreased, which matched the phenotype of osteopenia. It’s worth reminding that osteopenia was the final status resulted from overall effect of bone homeostasis, including reduced bone formation or/and enhanced bone resorption. It was reported SIRT6 deficiency could significantly reduce bone formation by hampering osteoblast differentiation^12,13^. Here, we focused on the other side and demonstrated that SIRT6 deficiency could also promote bone resorption via increasing osteoclast differentiation and activation both *in vivo* and *in vitro*.

One of the important physiological roles of SIRT6 is transcriptional repression^8,18^. As to focus on bone resorption in this study, the hyperactive NF-κB signaling is too closely relevant and noticeable to avoid being investigated as the following reasons. (1) It has been revealed that SIRT6 attenuates NF-κB signaling via interacting with the NF-κB RelA subunit and deacetylates histone H3 lysine 9 (H3K9) at NF-κB target gene promoters^16^. It might be the hyperactive NF-κB signaling that is responsible for the progeria phenotypes of mutant mice, which include bone loss^16,18,19^. (2) It is convincingly substantiated that NF-κB signaling is an essential factor for osteoclast differentiation, especially in osteoclast activation for bone resorption^17,20^. (3) NF-κB signaling is well-known as a transcription factor that plays critical role in regulating inflammation^21,22^. And it also has been revealed that SIRT6 deficiency is involved in chronic inflammation^19,23,24^. Inflammation is characterized by the activation of several cell populations that produce inflammatory cytokines, including NF-κB, IL-6, TNF-α, etc^25–27^. And most of the inflammatory cytokines are more or less involved in osteoclastogenesis^17,25–28^. Therefore, it has been substantiated inflammation could lead to both local and systemic excessive bone resorption^29,30^. For all these reasons, here we detected the expression of NF-κB signaling exclusively in bone. Expectedly, NF-κB signaling in bone of SIRT6 null mice was significantly activated. Taken together, our data, consistent with many previous related findings mentioned above, suggested that SIRT6 deficiency resulted in enhanced NF-κB signaling, which might lead to excessive osteoclast activation and hyperactive bone resorption in turn.

Osteoclast is differentiated from BMMs with the stimulation of receptor activator of nuclear factor κB ligand (RANKL) and macrophage colony stimulating factor (MCSF) *in vitro*^3^. At the early stage of osteoclastogenesis, BMMs actively proliferate with the stimulation of MCSF^31–33^. On the one hand, it’s reported the cell density, the need of securing a sufficient number of pre-osteoclast, is a critical determinant for the formation of mature osteoclast^32,33^. Thus, any genetic or pharmacological intervention that affected the number of pre-osteoclast could have a substantial impact on the number of multinuclear-fused osteoclast, thus affecting the osteoclast differentiation^33^. On the other hand, in this work we found out that SIRT6 deficiency could promote the proliferation of osteoclast precursors in the presence of MCSF at the early stage of osteoclast-inducing culture. In fact, the proliferation of various cells, not just BMMs or osteoclast precursors, could be promoted by SIRT6 deficiency and suppressed by SIRT6 overexpression^12,34,35^. Thus, it seemed that SIRT6 had a general role in cell proliferation. Based on these findings, our results suggested that SIRT6 deficiency could increase osteoclast, at least partially resulted from the enhanced proliferation of SIRT6 null osteoclast precursors.

During the osteoclast formation *in vitro*, HSC (haematopoietic precursors) or CFUM (colony forming unit macrophage) differentiate into monocytes/macrophage with MCSF induction, and then differentiate into multinuclear-fused osteoclast (multi-nucleus and TRAP^+^), and then multinuclear giant osteoclast as the final status^3,36^. During this process, the sizes of these cells gradually become larger. From this aspect only, without considering any other genetic or pharmacological intervention, it seems the larger cells should have stronger bone resorbing capacity. However, to the best of our knowledge, we failed to find out the definite or strong corresponding relation between the size and the bone resorbing capacity of osteoclast. According to our works, we believe the number of osteoclast (of course, which should be multinuclear and TRAP^+^) may be more important to account for bone resorbing capacity, compared with the size of osteoclast^31,32^. Additionally, the smaller osteoclast of SIRT6 deficiency in the cell culture was not like the smaller osteoclast of wild type, since it pathologically changed. So, our data also can’t draw a conclusion that smaller osteoclast of wild type has stronger bone resorbing capacity. Together, our results suggested SIRT6 deficiency could result in smaller but more number of osteoclast with multinuclear-fused and TRAP^+^ *in vitro*, which resulted in higher bone resorption in turn.

Osteoclast differentiation needs a variety of factors, including several key cytokines produced by osteoblast^3^. That is to say, the process of osteoclast differentiation needs osteoblast as inducing support system^25,36^. Reports have confirmed that SIRT6 could regulate osteoblast proliferation and differentiation^12,13^. Thus, we taken one step further to figure out the cellular way, SIRT6 regulated osteoclast, was through osteoblast paracrine or osteoclast-autonomous. So, the osteoblast/osteoclast co-culture was performed, and which undoubtedly could better simulate the physiological process of osteoclast differentiation with osteoblast crosstalk, compared with the RANKL-stimulated osteoclast culture *in vitro*^36,37^. Our data showed it was osteoblast, compared with the osteoclast/osteoclast precursors of SIRT6 deficiency, that made more contribution to increasing osteoclast formation. Thus, our results strongly suggested the cellular pathway, SIRT6 deficiency promoted osteoclast formation, was mainly through osteoblast paracrine manner, rather than osteoclast-autonomous behavior.

By comprehensively analyzing the findings from this study and other previous related studies^13–15^, we propose SIRT6 may influence osteoclastogenesis through several pathways synchronously, including osteoclast-autonomous mechanisms, osteoclast precursor proliferation and osteoblast paracrine. In fact, both of osteoclast precursor proliferation and osteoblast paracrine are as important as osteoclast-autonomous mechanisms to osteoclastogenesis, as discussed above. The bone-resorbing capacity of osteoclast must be influenced by the effect from both negative and positive regulations. It was reported SIRT6 could form a complex with Blimp1(B lymphocyteinduced maturation protein-1) to negatively regulate the expression of anti-osteoclastogenic gene^13,15^. It seemed SIRT6 deficiency negatively regulated osteoclastogenesis via osteoclast-autonomous mechanisms, which might be helpful to explain the smaller size of SIRT6 null osteoclast in cell culture, compared with the osteoclast in wild type. And this phenotype was also agreed with our results^14^. In this work, in order to focus on the bone resorption of SIRT6 null mice, we taken one step further to investigate the role of osteoclast precursor proliferation and SIRT6-null osteoblast in osteoclastogenesis. And we believed the overall activation of osteoclast was increased in SIRT6 null mice. Firstly, we found out SIRT6 deficiency could enhanced the number of osteoclast via promoting osteoclast precursor proliferation, which may be more important to the bone-resorbing capacity, compared with the size of osteoclast, as discussed above. Secondly, in consideration of the fact that the condition of osteoclast differentiation in cell-culture couldn’t directly reveal the definite bone-resorbing capacity of osteoclast, The Pit Resorption Assay we performed was the direct evidence for stronger bone-resorbing capacity of SIRT6 null osteoclast. Thirdly, since the osteoblast/osteoclast co-culture was certainly better to uncover the regulation on osteoclast in SIRT6 null mice, compared with the RANKL-stimulated osteoclast culture, it’s convincingly substantiated that it’s SIRT6-null osteoblast paracrine, rather than osteoclast-autonomous mechanisms, had a dominant role in osteoclastogenesis. Moreover, it’s reported that the expression of RANKL in bone cells was increased in SIRT6-null mice, while the expression of Opg, as well as Opg/RANKL, was decreased in SIRT6-null mice^38^. It strongly suggested osteoclastogenesis was enhanced by SIRT6-null osteoblast paracrine. Fourthly, it shouldn’t ignore the condition of hyperactive NF-κB signaling and systemic inflammation in SIRT6 null mice, which had been convincingly substantiated in previous works as discussed above^9,16^, and which should promote bone resorption. Together, it seemed that SIRT6 deficiency might negatively regulate osteoclastogenesis via osteoclast-autonomous mechanisms, but positively regulate osteoclastogenesis via impacting osteoblast paracrine and promoting osteoclast precursor proliferation as well. Although the details of regulation mechanisms still need to be further investigated, we believe SIRT6 deficiency promotes the bone resorption in SIRT6 null mice, which results from the overall excessive effect of osteoclast.

## Conclusions

Our work revealed that the osteoclast activity in SIRT6 null mice was significantly increased both *in vivo* and *in vitro*. The enhanced osteoclast activity certainly led to hyperactive bone resorption, and partially accounted for osteopenia in turn. Our results further suggested that (1) the upregulated NF-κB signaling, triggered by SIRT6 deficiency, might play a key role in elevating osteoclast activation, and (2) the enhanced osteoclast at least partially resulted from the enhanced proliferation of osteoclast precursors, and (3) SIRT6 deficiency promoted osteoclast formation mainly through impacting on osteoblast paracrine behavior at the cellular level, compared with osteoclast-autonomous regulation.

## Materials and Methods

### Animals and Histomorphometric Analysis

All the protocols were reviewed and approved by the Institutional Animal Care and Use Committees at Sichuan University. All experiments were carried out in accordance with the guidelines and regulations of the Institutional Animal Care and Use Committees at Sichuan University. SIRT6 knockout mice were generated as described by the Jackson Laboratory (JAX^®^ Mice and Services, 006050, Bar Harbor, ME, USA), and the genotyping was followed the instructions of the Jackson Laboratory. The homozygous SIRT6 mice (129/SvJ) were bred from the pairs of heterozygous SIRT6 mice. 3-week old male mice were used for this study. Bone histomorphometric analysis was conducted at State Key Laboratory of Oral Diseases, Sichuan University. For μ-CT analysis, the third lumbar vertebras and the femurs were scanned using μ-CT Scanner (μ-CT50, Scanco, Bassersdorf, Zurich, Switzerland), operated at 60 kV, 165 μA, 450 ms exposure time and 10-μm resolution. We used standardized nomenclature for the bone parameters measured according to the μ-CT Scanner protocol.

### Histology Analysis

Paraffin-embedding and sectioning methods were used to prepare histological sections. For Alcian Blue-Alizarin Red Skeletal Staining, mouse samples were fixed with 4% paraformaldehyde for 48 h, and then putted into 100% ethanol for 24 h, and then stained in Alcian Blue solution consisting of 0.015% Alcian Blue 8GX (A5268, Sigma, St. Louis, USA), 20% acetic acid and 70% ethanol for 24 h. After rinsed in water twice, the samples were stained in alizarin red solution consisting of 0.025% alizarin red (A5533, Sigma, St. Louis, USA) and 3% KOH for 24 h. Finally, the samples were washed in tap water and photoed. Von Kossa and Van Gieson staining was performed as following, the sample slides, prepared by hard tissue slicing, were dipped in Von Kossa staining solution (10 mg/ml of argentum nitricum, CAS:7761-88-8, Kelong, Chengdu, China) for 10 min, and exposed to ultraviolet lamp for about 90 min, and then dipped in Van Gieson solution (25 mg/mlpicric acid, CAS:88-89-1, Kelong, Chengdu, China) for 10 min, finally washed twice in tap water and photoed. For Calcein Blue and TRAP staining, the sample slides were covered with pre-incubation solution (50 mM sodium acetate CAS: 6131-90-4, Kelong, Chengdu, China, and 30 mM sodium nitrite, CAS:7632-00-0, Kelong, Chengdu, China) for 10 min. The slides were then drained and covered with the buffer containing 100 µM ELF^®^97 (E6589, Life Tech, USA) phosphatase substrate, and followed by UV exposure for about 60 min at room temperature, and then submerged in Calcein Blue solution (M1255, Sigma, St. Louis, USA, 60 mM in PBS) for 30 min. The reaction was stopped by submerging the slides in tap water for 10 min, and the slides were sealed with resinene (Solarbio Science and Technology Co., Ltd. Beijing, China) and photoed. TRAP and Aniline Blue staining were performed using a Leukocyte Acid Phosphatase Kit (#387 A, Sigma-Aldrich, St. Louis, USA) and counterstained with Aniline Blue solution (B8563, Sigma, St. Louis, USA). TRAP-positive multinuclear cells were counted under microscopic examination. The statistical analysis was calculated by using Image-Pro Plus software (IPP version 6.0, Media Cybernetics, Carlsbad, CA, USA) and the bar graph were plotted by using Graphpad Prism 4.0 (GraphPad Software, San Diego, California,USA).

### Enzyme-linked Immunosorbent Assay (ELISA)

Enzyme-linked immunosorbent assay for ACP5 was performed according the manufacturer’s protocol (Catalogue No. SEA902Mu, Cloud Clone, Wuhan, China). The mouse serums were detected. Briefly, 96-well plates coated with ACP5 antigen were incubated with appropriately diluted serum samples, and then ACP5 was detected by using anti-human IgG-HRP conjugate and o-phenylenediamine substrate at 450 nm of optical density (OD) of a microplate reader (Bio-Tek Co., Bedfordshire, UK). The OD value of the serum sample, obtained on antigen positive well, was subtracted from that on the antigen negative well. Sample concentrations were measured against the standard curve plotted by using a series of known concentrations of standards in manufacturer’s kit.

### Cell Culture and Analysis

Bone marrow haematopoietic cells, collected from long bone marrow, were cultured (0.3 × 10^5^ bone marrow cells per 48-well) and induced into osteoclast in α-modified Eagle’s minimal essential medium (α-MEM; Catalogue No. SH30265; Hyclone Laboratories, Logan, UT, USA) with the presence of MCSF (100 μg/ml, SRP3221, Sigma, St. Louis, USA) and RANKL (100 μg/ml, R0525, Sigma, St. Louis, USA) for about 7–9 days, which was also supplemented with 20% fetal bovine serum (FBS, Hyclone Laboratories, Logan, UT, USA) and 1% penicillin/streptomycin (P/S, Life Technologies, Gaithersburg, MD, USA). Then the cells were fixed with 4% paraformaldehyde for 35 min and then stained for tartrate-resistant acid phosphatase. TRAP-positive multinuclear cells were counted under microscopic examination. For bone Pit Resorption Assay, bovine cortical bone slices were sterilely prepared and placed into the bottom of cell-wells and then cell culture was carried out as described above. After that, the slices were clearly washed and then taken images by scanning electron microscope (SEM: S-4700, Hitachi Co, Tokyo, Japan). The absorbed areas were calculated by using the Image-Pro Plus software (IPP version 6.0, Media Cybernetics, Carlsbad, CA, USA). For CCK-8 Test, Cells were cultured for 24 h in the presence of MCSF, then treated with 10% CCK-8 solution (Biyuntian Biotechnology, Jiangsu, China) for 2 h at 37 °C. The optical density was then measured at 450 nm by using a microplate reader (Bio-Tek Co., Bedfordshire, UK). For osteoblast/osteoclast co-culture, the calvarial bone from new born mice was prepared for osteoblast culture at 5 × 10^4^ cells per 48-well. Calvaria was cuted into pieces and then digested in PBS (pH = 7.4) containing 0.5% trypsin for 60 min, and then transferred into α-modified Eagle’s minimal essential medium (α-MEM; Catalogue No. SH30265; Hyclone Laboratories, Logan, UT, USA) with 20% fetal bovine serum (FBS, Hyclone Laboratories, Logan, UT, USA) and 1% penicillin/streptomycin (P/S, Life Technologies, Gaithersburg, MD, USA). When the osteoblast density reached 95% confluence per well, bone marrow haematopoietic cells (0.3 × 10^5^ bone marrow cells per 48-well) were gently placed on the top of osteoblast for osteoclast differentiation in α-MEM medium with 20%FBS, 1%P/S, 10^−8^ M 1,25-dihydroxyvitamin D3 (D1530, Sigma, St. Louis, USA) and 10^−6^ M prostaglandin E2 (P0409, Sigma, St. Louis, USA) for about 13–15 days.

### Gene Expression Analysis

Total mRNA was extracted with TRIzol reagent (Cat#15596018, Invitrogen, Carlsbad, CA, USA) according to the manufacturer’s protocol. mRNA was treated with DNase and then used for first-strand cDNA synthesis via oligo (dT) primer and SuperScript II reverse transcriptase (Cat#11756050, Invitrogen, Carlsbad, CA, USA). Quantitative real-time PCR was performed in quadruplicate by using SYBR Green Supermix on the Real-Time Detection System (Bio-Rad Laboratories, Hercules, CA, USA). The relative expression of mRNA was normalized with GAPDH as internal reference by using the 2^−∆∆^ CT method.

### Statistics

Data are presented as mean ± S.D. from at least three independent experiments. Statistical differences were calculated by two-tailed student’s t-test of Microsoft Excel software. P < 0.05 was considered statistically significant.

### Data Availability

All data generated or analysed during this study are included in this published article and its Supplementary Information file.

## Electronic supplementary material

Supplementary Information

## References

[1] Ebeling PR (2013). Osteoporosis in men. Curr Opin Rheumatol.

[2] McClung M, Baron R, Bouxsein M (2017). An update on osteoporosis pathogenesis, diagnosis, and treatment. Bone.

[3] Feng X, Teitelbaum SL (2013). Osteoclasts: New Insights. Bone Res.

[4] Loi F (2016). Inflammation, fracture and bone repair. Bone.

[5] Adler RA (2014). Osteoporosis in men: a review. Bone Res.

[6] Jobke B, Milovanovic P, Amling M, Busse B (2014). Bisphosphonate-osteoclasts: changes in osteoclast morphology and function induced by antiresorptive nitrogen-containing bisphosphonate treatment in osteoporosis patients. Bone.

[7] Marcoline FV, Ishida Y, Mindell JA, Nayak S, Grabe M (2016). A mathematical model of osteoclast acidification during bone resorption. Bone.

[8] Gertler AA, Cohen HY (2013). SIRT6, a protein with many faces. Biogerontology.

[9] Mostoslavsky R (2006). Genomic instability and aging-like phenotype in the absence of mammalian SIRT6. Cell.

[10] Wu Y (2015). Overexpression of Sirtuin 6 suppresses cellular senescence and NF-kappaB mediated inflammatory responses in osteoarthritis development. Sci Rep.

[11] Lee HS (2013). Overexpression of sirtuin 6 suppresses inflammatory responses and bone destruction in mice with collagen-induced arthritis. Arthritis Rheum.

[12] Sun H, Wu Y, Fu D, Liu Y, Huang C (2014). SIRT6 regulates osteogenic differentiation of rat bone marrow mesenchymal stem cells partially via suppressing the nuclear factor-kappaB signaling pathway. Stem Cells.

[13] Sugatani T, Agapova O, Malluche HH, Hruska KA (2015). SIRT6 deficiency culminates in low-turnover osteopenia. Bone.

[14] Zhang DM (2016). Phenotypic research on senile osteoporosis caused by SIRT6 deficiency. Int J Oral Sci.

[15] Park SJ (2016). Sirt6 cooperates with Blimp1 to positively regulate osteoclast differentiation. Sci Rep.

[16] Kawahara TL (2009). SIRT6 links histone H3 lysine 9 deacetylation to NF-kappaB-dependent gene expression and organismal life span. Cell.

[17] Soysa NS, Alles N (2009). NF-kappaB functions in osteoclasts. Biochem Biophys Res Commun.

[18] Lombard DB, Schwer B, Alt FW, Mostoslavsky R (2008). SIRT6 in DNA repair, metabolism and ageing. J Intern Med.

[19] Vitiello M (2017). Multiple pathways of SIRT6 at the crossroads in the control of longevity, cancer, and cardiovascular diseases. Ageing Res Rev.

[20] Swarnkar G, Abu-Amer Y (2015). Regulation of NF-kappaB signaling in osteoclasts and myeloid progenitors. Methods Mol Biol.

[21] Adler AS, Kawahara TL, Segal E, Chang HY (2008). Reversal of aging by NFkappaB blockade. Cell Cycle.

[22] Sun SC (2017). The non-canonical NF-kappaB pathway in immunity and inflammation. Nat Rev Immunol.

[23] Xiao C (2012). Progression of chronic liver inflammation and fibrosis driven by activation of c-JUN signaling in Sirt6 mutant mice. J Biol Chem.

[24] Kugel S, Mostoslavsky R (2014). Chromatin and beyond: the multitasking roles for SIRT6. Trends Biochem Sci.

[25] Boyce BF, Rosenberg E, de Papp AE, Duong LT (2012). The osteoclast, bone remodelling and treatment of metabolic bone disease. Eur J Clin Invest.

[26] Skelly DT, Hennessy E, Dansereau MA, Cunningham C (2013). A systematic analysis of the peripheral and CNS effects of systemic LPS, IL-1beta, TNF-alpha and IL-6 challenges in C57BL/6 mice. PLoS One.

[27] Feng W (2017). Combination of IL-6 and sIL-6R differentially regulate varying levels of RANKL-induced osteoclastogenesis through NF-κB, ERK and JNK signaling pathways. Scientific Reports.

[28] Johnson RW (2015). Glycoprotein130 (Gp130)/interleukin-6 (IL-6) signalling in osteoclasts promotes bone formation in periosteal and trabecular bone. Bone.

[29] Redlich K, Smolen JS (2012). Inflammatory bone loss: pathogenesis and therapeutic intervention. Nat Rev Drug Discov.

[30] Goldring SR (2015). Inflammatory signaling induced bone loss. Bone.

[31] Tanaka S (1993). Macrophage colony-stimulating factor is indispensable for both proliferation and differentiation of osteoclast progenitors. J Clin Invest.

[32] Motiur Rahman M (2015). Proliferation-coupled osteoclast differentiation by RANKL: Cell density as a determinant of osteoclast formation. Bone.

[33] Ikeda K, Takeshita S (2016). The role of osteoclast differentiation and function in skeletal homeostasis. J Biochem.

[34] Cea M (2016). Evidence for a role of the histone deacetylase SIRT6 in DNA damage response of multiple myeloma cells. Blood.

[35] Nagai K (2015). Depletion of SIRT6 causes cellular senescence, DNA damage, and telomere dysfunction in human chondrocytes. Osteoarthritis Cartilage.

[36] Deng L (2015). Osteoblast-derived microvesicles: A novel mechanism for communication between osteoblasts and osteoclasts. Bone.

[37] Chen X (2017). Osteoblast-osteoclast interactions. Connect Tissue Res.

[38] Mu, W. *et al*. Metformin promotes the proliferation and differentiation of murine preosteoblast by regulating the expression of sirt6 and oct4. *Pharmacol Res*, 10.1016/j.phrs.2017.11.020 (2017). 10.1016/j.phrs.2017.11.020 29162538

