# Supplementary material for: Evidence for excessive osteoclast activation in SIRT6 null mice
Source: Sci Rep. 2018 Jul 20;8:10992. doi: 10.1038/s41598-018-28716-z (PMC6054613; doi:10.1038/s41598-018-28716-z)
Supplement: Supplementary file 1 — Supplementary Information [file 41598_2018_28716_MOESM1_ESM.pdf]

Evidence for excessive osteoclast activation in SIRT6 null mice

Demao Zhang[#1], Junjun Jing[#1], Feng Lou[#1], Ruimin Li[2], Yilin Ping[3], Fanyuan Yu[1], Fanzi Wu[1], Xiao Yang[1], Ruoshi Xu[1], Feifei Li[1], Ke Wang[4], Mingru Bai[1], Caixia Pi[1], Jing Xie[*1], Liwei Zheng[1], Ling Ye[1], Xuedong Zhou[*1]

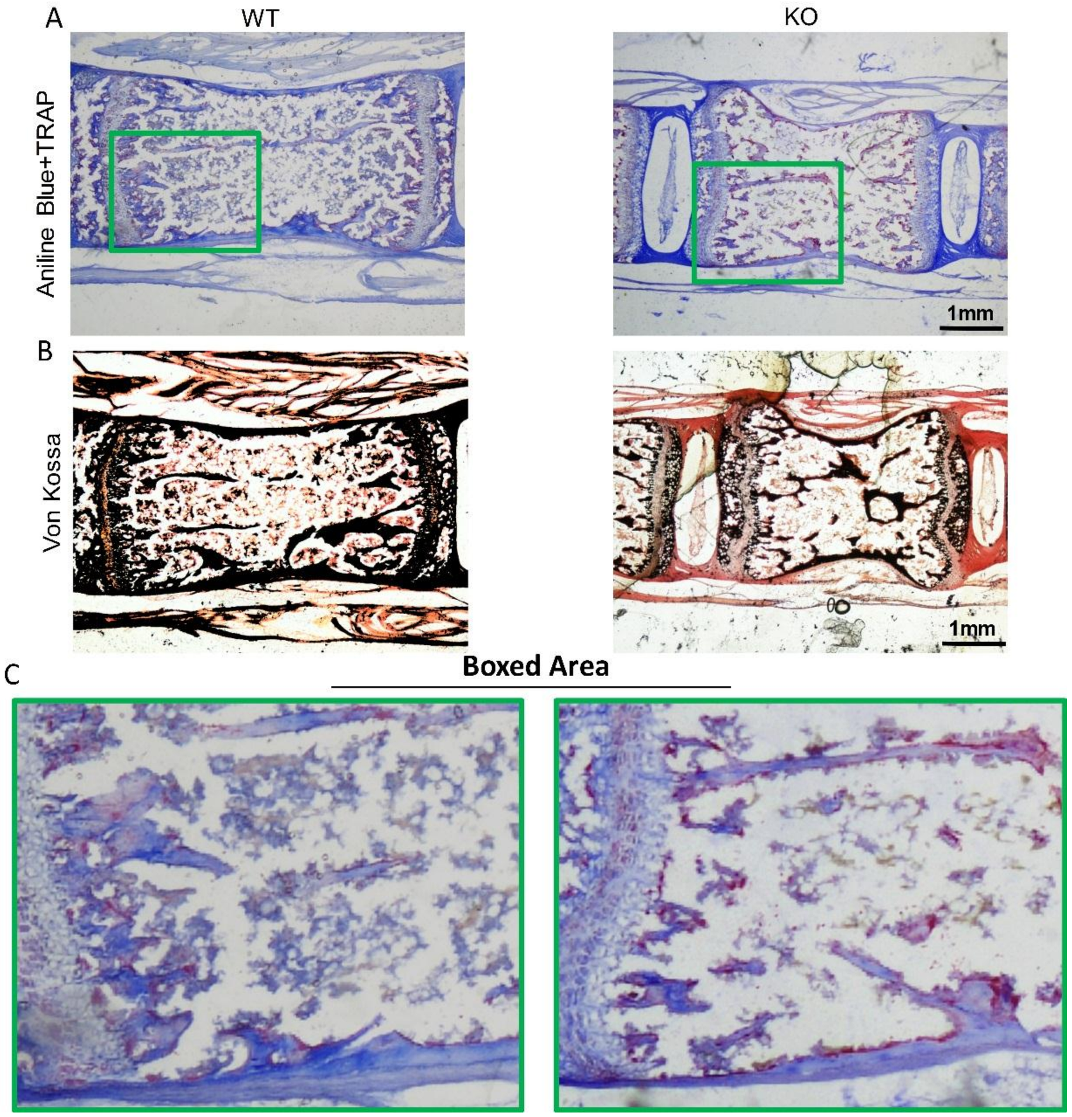

Figure S1: SIRT6 deficiency resulted in more osteoclast and less bone mass in the tail of SIRT6 KO mice *in vivo*. **(A)** Trap stain showed the enhanced osteoclast formation (red) in the tail of SIRT6 KO mice. Aniline Blue, the background stain (blue). **(B)** Von Kossa stain showed the reduced bone mass (black) in the tail of SIRT6 KO mice. Van Gieson stain, the background stain (red). **(C)** The boxed area from (A) indicated the enhanced osteoclast in the KO group.

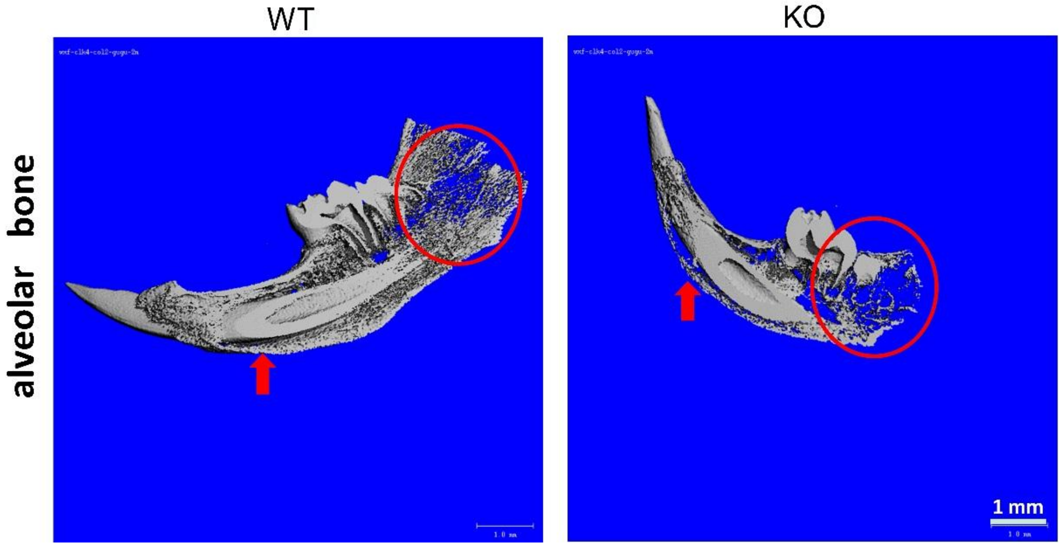

Figure S2: SIRT6 deficiency resulted in alveolar bone loss *in vivo.* μ-CT scanning showed the bone mass of alveolar bone sharply decreased in the SIRT6 KO mice, compared to the littermates of WT.

Primers	Sequence 5' --> 3'
**Wild type**	GTCACTCTGTCCTCTTGGTA
**Common**	CGCTTCCCGATGTTTCA
**Mutant**	GCA ATA GCA TCA CAA ATT TCA C

Table S1: Primers list for SIRT6 genotyping.

Primers	Sequences 5' --> 3'
**RANKL Fwd**	GTCACTCTGTCCTCTTGGTA
**RANKL Rev**	CGCTTCCCGATGTTTCAT
**M-CSF Fwd**	CTGGAAGGAGGATCAGCAAG
**M-CSF Rev**	ATGTCTGAGGGTTTCGATGG
**MMP9 Fwd**	AGTTGCCCCTACTGGAAGGT
**MMP9 Rev**	GTGGATAGCTCGGTGGTGTT
**NEMO Fwd**	GGCGGCACGTTTTACTCTTT
**NEMO Rev**	CCGTCTCCAGGAGGTTAATGC
**ICAM-1N Fwd**	CCGCAGGTCCAATTCACACT
**ICAM-1N Rev**	CAGAGCGGCAGAGCAAAAG
**C/EBPα Fwd**	CAAGAACAGCAACGAGTACCG
**C/EBPα Rev**	GTCACTGGTCAACTCCAGCAC
**i-NOS Fwd**	CTCACTGGGACAGCACAGAA
**i-NOS Rev**	GATGTGGCCTTGTGGTGAA
**Itgam Fwd**	AGTGCTGGGAGACGTGAATG
**Itgam Rev**	GCACTGAGGCTGGCTATTGA
**Rank Fwd**	TGGGTGATTTTCTTTTGGTGGG
**Rank Rev**	CCAAGAACCAGTGCTCGTGA

Table S2: Primers list for qPCR.
